# S^2^ALM: Sequence-Structure Pre-trained Large Language Model for Comprehensive Antibody Representation Learning

**DOI:** 10.34133/research.0721

**Published:** 2025-08-19

**Authors:** Mingze Yin, Hanjing Zhou, Jialu Wu, Yiheng Zhu, Yuxuan Zhan, Zitai Kong, Hongxia Xu, Chang-Yu Hsieh, Jintai Chen, Tingjun Hou, Jian Wu

**Affiliations:** ^1^ College of Computer Science and Technology, Zhejiang University, Hangzhou, China.; ^2^ Institute of Wenzhou, Zhejiang University, Wenzhou, China.; ^3^ Zhejiang Cuisine Bird Supply Chain Management Co., Ltd., Hangzhou, China.; ^4^ College of Pharmaceutical Sciences, Zhejiang University, Hangzhou, China.; ^5^ School of Public Health, Zhejiang University, Hangzhou, China.; ^6^ AI Thrust, Information Hub, HKUST(GZ), Guangzhou, China.; ^7^ Zhejiang Key Laboratory of Medical Imaging Artificial Intelligence, Hangzhou, China.

## Abstract

Antibodies safeguard our health through their precise and potent binding to specific antigens, demonstrating promising therapeutic efficacy in the treatment of numerous diseases, including COVID-19. Recent advancements in biomedical language models have shown the great potential to interpret complex biological structures and functions. However, existing antibody-specific models have a notable limitation that they lack explicit consideration for antibody structural information, despite the fact that both 1-dimensional sequence and 3-dimensional structure carry unique and complementary insights into antibody behavior and functionality. This paper proposes the **S**equence-**S**tructure multi-level pre-trained **A**ntibody **L**anguage **M**odel (S^2^ALM), combining holistic sequential and structural information in one unified, generic antibody foundation model. We construct a hierarchical pre-training paradigm incorporated with 2 customized multi-level training objectives to facilitate the modeling of comprehensive antibody representations. S^2^ALM’s representation space uncovers inherent functional binding mechanisms, biological evolution properties, and structural interaction patterns. Pre-trained over 75 million sequences and 11.7 million structures, S^2^ALM can be adopted for diverse downstream tasks: accurately predicting antigen–antibody binding affinities, precisely distinguishing B cell maturation stages, identifying antibody crucial binding positions, and specifically designing novel coronavirus-binding antibodies. Remarkably, S^2^ALM outperforms well-established and renowned baselines and sets new state-of-the-art performance across extensive antibody-specific understanding and generation tasks. S^2^ALM’s ability to model comprehensive and generalized representations further positions its potential to advance real-world therapeutic antibody development, potentially addressing unmet academic, industrial, and clinical needs.

## Introduction

Antibodies, also known as immunoglobulins, are specialized proteins produced by the immune system to protect against a range of diseases (e.g., severe acute respiratory syndrome coronavirus 2 [SARS-CoV-2]), which fulfill the responsibility as guardians in the human body via evolving to complement their structures with the corresponding antigen structures [1–4]. Due to the high specificity and low adverse effect of antibodies, antibody drugs have important clinical significance and account for nearly one-fifth of new drug approvals by the Food and Drug Administration each year [5]. By mimicking the actions of the immune system, these drugs specifically target harmful agents like viruses and cancer cells, to detect viral infections or stimulate T cell immunity in cancer treatment [6–9]. Therefore, deciphering the information stored in antibody sequences and structures is crucial for understanding immune responses and disease development, and may accelerate the development of therapeutic antibodies for broad-spectrum disease treatment.

To alleviate the burden of time-consuming wet-lab experiments, in recent years, the computer-aided antibody engineering has emerged to improve the efficiency of antibody evolution and identify promising therapeutic antibodies with desirable developability profiles. While interpretable, traditional representation learning approaches depend on inefficient hand-crafted features that may miss hidden or latent patterns in diverse data. Drawing inspiration from natural language processing with the introduction of pre-trained foundational models, similar artificial intelligence technologies have emerged as the pivotal technology to interpret the language of biology [10–14]. Pre-trained on large-scale antibody corpora, antibody-specific large language models (ALMs) [6,7,15–18] have exhibited powerful capabilities in advancing the understanding of antibody structures and functions. Additionally, ALMs consistently outperform general-purpose protein language models (PLMs) because the mechanism of antibody evolution is strongly biased to the target antigen. This finding inspires us to dive deep into the development of comprehensive antibody-specific pre-training.

**Fig. 1.**
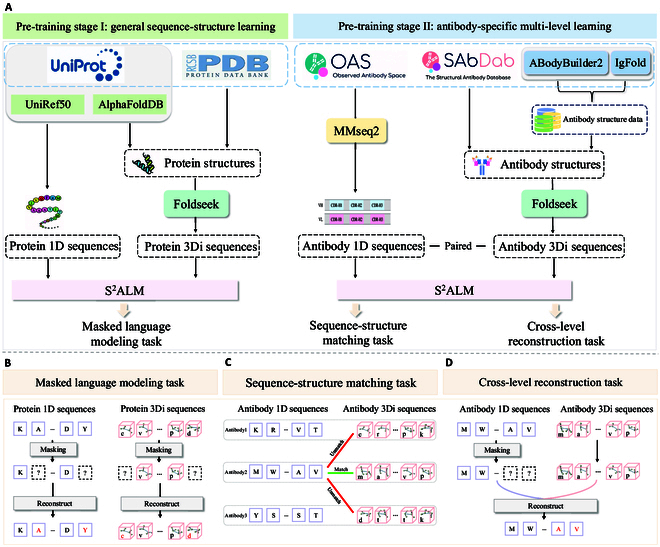
Overview of the proposed hierarchical pre-training paradigm containing 2 stages. (A) In stage I, S^2^ALM aims at general sequence-structure learning with protein sequences and structures. In stage II, S^2^ALM learns antibody-specific multi-level knowledge using antibody sequences and structures. (B) Masked language modeling (MLM) reconstructs the masked tokens based on the contextualized information. (C) Sequence-structure matching (SSM) identifies the matching relationships between 1D and 3Di sequences. (D) Cross-level reconstruction (CLR) reconstructs the corrupted tokens based on hybrid information from both 1D and 3Di sequences.

The central tenet of molecular biology is that an antibody’s amino acid sequence determines its 3-dimensional (3D) structure, and the 3D structure determines its biological function. The spatial structure plays an integral role for antibody functional characterization, as the principle of antigen–antibody structural binding indicates that antibody structures directly determine biological functions. Consequently, injecting structural information into antibody pre-training emerges a highly compelling endeavor. In this paper, we propose the **S**equence-**S**tructure multi-level pre-trained **A**ntibody **L**anguage **M**odel (S^2^ALM), incorporating antibody sequential and structural information to construct a comprehensive antibody foundation model. To accomplish this, we collect a holistic pre-training dataset with massive 1D sequences and 3D structures, and propose a hierarchical pre-training paradigm (Fig. 1), accompanied by 2 customized training objectives: sequence-structure matching (SSM) and cross-level reconstruction (CLR). These methodologies allow the model to concurrently process and analyze antibody sequence and structure data, further modeling comprehensive antibody representations to facilitate the deciphering of the biological language.

Through extensive interpretability analyses, S^2^ALM exhibited an emergent understanding of unobserved patterns inherent in antibody structures and functions. Furthermore, to illustrate S^2^ALM ’s practical effectiveness, we conducted in-depth examinations to assess the performance of S^2^ALM across a range of downstream scenarios for antibody analysis. The outstanding performance verifies the S^2^ALM’s substantial potential to serve as the novel antibody foundation model and highlights its applicability in real-world therapeutic development.

The main contributions of S^2^ALM are 3-fold:

• Pre-trained on a large-scale multi-level dataset containing 75 million 1D sequences and 11.7 million 3D structures from protein and antibody domains, S^2^ALM learns comprehensive antibody representations uncovering inherent patterns within structures and functions.
• A hierarchical pre-training paradigm incorporated with 2 customized training objectives, sequence-structure matching and cross-level reconstruction, is proposed to promote the injection of antibody structural information.
• S^2^ALM consistently exceeds state-of-the-art performance on extensive tasks including antigen binding prediction, B cell maturation analysis, antibody paratope prediction, binding affinity prediction, and computational antibody design, exhibiting its broad applicability in therapeutic antibody development and immune response analysis.

## Results

### S^2^ALM: pre-trained ALM integrating sequences and structures

In this paper, we pre-trained a large-scale antibody-specific language model S^2^ALM, which integrates multi-level information of 1D sequences and 3D structures for comprehensive antibody representation learning. To accomplish this, the model architecture is built upon ESM-2 [19], comprising 650 million trainable parameters to facilitate the large-scale pre-training. Additionally, we collect a large-scale comprehensive training dataset, composed of sequences and structures from both protein and antibody domains. Furthermore, Foldseek [20] is introduced to execute the efficient encoding of protein and antibody 3D structures, transforming 3D structures to 3Di sequences. Given sufficient 1D and 3Di sequences of proteins and antibodies, we propose a hierarchical pre-training paradigm, where 2 pre-training stages are constructed to foster holistic understandings of general proteins and specific antibodies correspondingly. To further combine sequential and structural information, we develop 2 customized pre-training objectives, namely, sequence-structure matching and cross-level reconstruction. Equipped with a comprehensive dataset, an efficient encoding technique, a hierarchical pre-training paradigm, and tailored pre-training objectives, the resulting S^2^ALM acquires general-purpose antibody representations containing biological information derived from both 1D sequences and 3D structures. To our best knowledge, S^2^ALM is the first large-scale pre-trained antibody-specific language model that encodes sequences and structures in a hybrid manner for comprehensive representation learning. In contrast to ALMs solely trained on massive sequences, our main contribution to the biological community lies in the additional infusion of structural information into S^2^ALM. (See detailed advantages over other large language models [LLMs] in Note S4.) The structure-enhanced representations demonstrate a strong potential to broadly benefit antibody understanding and generation applications.

### Observation of multi-scale organization in antibody representations

The variation observed in large antibody sequence datasets is influenced by processes at many scales, including functional properties that affect binding to specific antigens, species selection biases, and isotype biases that reflect different B cell maturation stages. Uncovering inherent patterns and properties is crucial for comprehending antibody properties. Unsupervised learning captures latent factors that, while unobserved, prove instrumental in elucidating the biological sequence variation [9]. We investigate the representation space of the pre-trained large language models at aforementioned multiple scales to look for signatures of biological organization.

For a more intuitive comparison, the t-SNE algorithm [21] is employed to visualize the antibody representations generated by the last layer of LLMs. To comprehensively evaluate the effectiveness of large-scale pre-training, it is necessary to compare representations encoded by the untrained S^2^ALM with randomly initialized weights and the pre-trained S^2^ALM [7]. We also include the well-established and powerful ESM-2 [19] (pre-trained on protein sequences) for comparison to further verify the superiority of the constructed S^2^ALM.

#### Pre-training encodes functional specificity

Antibodies have emerged as essential therapeutic agents in the treatment of various autoimmune, infectious, and metabolic diseases, mainly owing to their ability to specifically bind to corresponding antigens [6,22]. Precise identification of antibody functional specificity will greatly enhance the progress of antibody development and optimization. From the Observed Antibody Space (OAS) database [23], we systematically identify and filter out 6,000 antibodies that exhibit specific binding affinities toward 3 distinct pathogens: HIV, Ebola virus, and SARS-CoV-2.

**Fig. 2.**
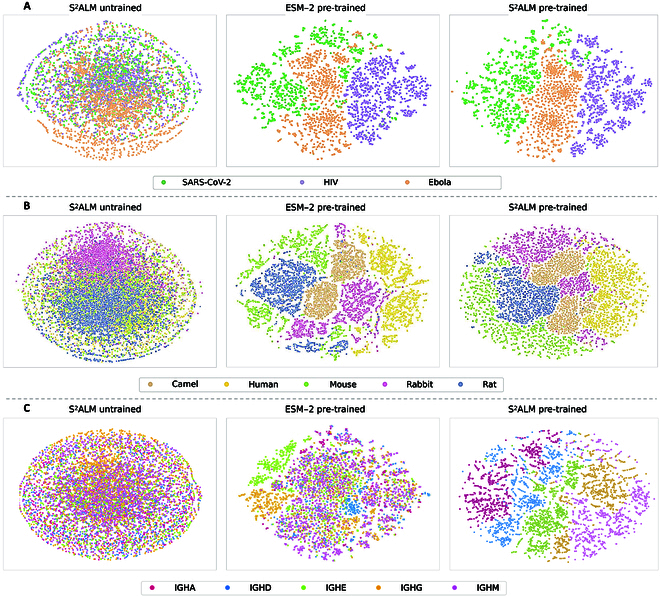
The t-SNE visualization results. Different colors indicate antibodies with different categories correspondingly. Untrained S^2^ALM and pre-trained ESM-2 [19] are included for comparison. The visualization analyses demonstrate that S^2^ALM contains information about functional specificity, biological species, and evolutionary isotypes in its comprehensive encoded representations.

We hereby present the t-SNE visualization analyses in Fig. 2A. Surprisingly, ESM-2 [19] somewhat succeeds in distinguishing among these 3 types of antibodies, but there remains some confusion regarding antibodies targeting SARS-CoV-2 and the Ebola virus. The pre-trained S^2^ALM produces more clearly aggregated antibody representations for all 3 pathogens, contrasting with scattered representations from the untrained model and ESM-2. This further verifies the necessity of our pre-training phase and indicates that the representations extracted by S^2^ALM contain antibody functional specificity information.

#### Pre-training encodes biological species

Due to substantial differences in their genetic information and immune function mechanisms, antibodies from different species are expected to exhibit notable distribution disparities. We randomly sample and collect 15,000 antibody sequences from the OAS database [23], ensuring an equal number of distinct species. The primary sources of biological species are taken into account for antibody sequencing, including camel, human, mouse, rabbit, and rat.

The t-SNE visualization is shown in Fig. 2B. We first observe that, when encoded by untrained S^2^ALM, all antibodies from different species tend to mix together in the t-SNE 2-dimensional space. Additionally, ESM-2 [19] successfully uncovers some clue about the species origins of various antibodies, whereas there are still some confounding issues regarding the boundaries of the clusters. Notably, antibodies of distinct species are nicely clustered together after being encoded by pre-trained S^2^ALM, indicating that the learned representations are encoded with rich biological species information.

#### Pre-training encodes evolutionary isotypes

Antibodies of different isotypes activate distinct effector mechanisms, manifesting at different stages of the immune response, and differing in structures and locations. Isotype expression reflects the maturation stage of a B cell. Naive B cells express immunoglobulin M (IgM) and IgD isotypes with unmutated variable genes, while expression of other antibody isotypes (IgG, IgA, and IgE) occurs after antigen exposure [24]. Distinguishing antibody isotypes evaluates the injection of evolutionary information during the model pre-training phase. We select 2,000 antibody sequences from the OAS database [23] per distinct isotype, resulting in a total of 10,000 sequences for t-SNE visualization analyses.

As illustrated in Fig. 2C, when exploiting representations extracted by untrained S^2^ALM and the pre-trained ESM-2 [19] for t-SNE projection, the organizations of antibodies with various isotypes are predominantly diffuse. This may be attributed to the fact that isotype classification heavily relies on antibody-specific evolutionary information, which the untrained S^2^ALM and the pre-trained ESM-2 are unable to capture. In contrast, we can observe a distinct clustering phenomenon utilizing antibody representations with different isotypes derived from the pre-trained S^2^ALM. The visualization provides a sanity check for the ability of S^2^ALM to extract the valuable information of antibody evolutionary isotypes.

### S^2^ALM captures clues on antibody structure

**Fig. 3.**
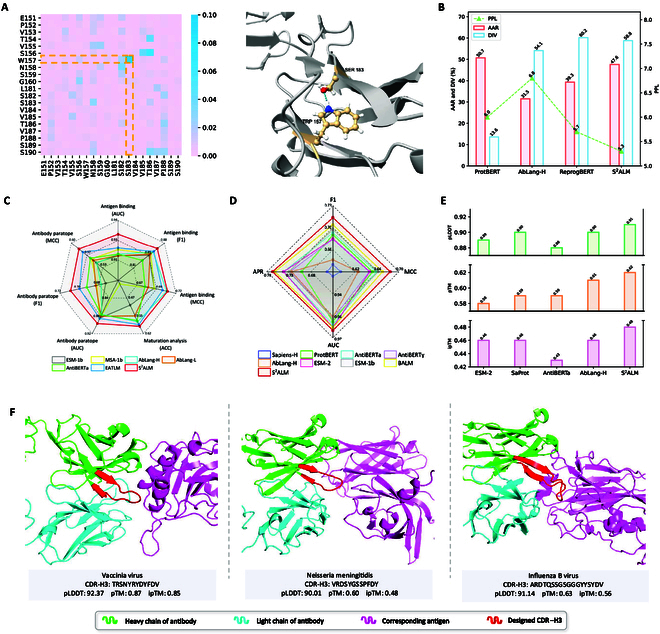
S^2^ALM exhibits superior performance on antibody understanding and generation tasks. (A) Interpretability analysis of S^2^ALM in capturing antibody structural interaction patterns. The heatmap reveals the self-attention values of the STE90-C11’s heavy chain, derived from the last hidden layer of the third head in S^2^ALM. The crystal structure of STE90-C11 (PDB: 7B3O) confirms the interaction mediated by the hydrogen bond between TRP157 and SER183. (B) Evaluation results of the generated antibodies on antibody CDR design task. S^2^ALM simultaneously balances the generative PPL, AAR, and DIV. (C and D) Experimental performance on antigen binding capability prediction, B cell maturation analysis, and antibody paratope prediction tasks. Antibody paratope prediction datasets are from Wang et al. [18] in (C) and Leem et al. [15] in (D). S^2^ALM consistently achieves state-of-the-art performance across all included evaluation metrics compared to all baseline models. (E) Structural evaluation results of the generated antibodies, utilizing AlphaFold3 [25] for 3D structure prediction. S^2^ALM surpasses other baseline models in terms of pLDDT, pTM, and ipTM. (F) 3D structure visualization of the generated antigen–antibody complexes. Targeting 3 specific pathogens (i.e., Vaccinia virus, *Neisseria meningitidis*, and Influenza B virus), S^2^ALM is employed to design the antibody CDR-H3 (highlighted in red). The stable and regular 3D structures of designed antigen–antibody complexes fully demonstrate the superiority of S^2^ALM .

It is essential for antibody-specific language models to identify and focus on crucial interaction sites during the representation learning process. The multi-head self-attention mechanism, which specifically focuses on the different aspects of antibodies, has the potential to capture sophisticated interdependencies within the antibody structure. As a practical demonstration of S^2^ALM’s proficiency in modeling the antibody structural interaction patterns, we execute a structural interpretability analysis, and we find that residue pairs with high self-attention scores can accurately reveal long-range structural contacts. Specifically, the antibody STE90-C11 (Protein Data Bank [PDB]: 7B3O), a SARS-CoV-2 neutralizing antibody that binds to the ACE2-RBD interface, is selected as an example input. The self-attention scores from the last hidden layer in S^2^ALM are extracted to build the heatmap, and we identify the potential hydrogen bond locations within the antibody structure. This allows assessing whether S^2^ALM identifies antibody residue structural interactions. As shown in Fig. 3A, the heatmap displays a high self-attention score between residues TRP157 and SER183 (highlighted in orange), serving as an indicator of potential structural associations among these 2 residues. Correspondingly, the intricate crystal structure precisely substantiates the existence of a hydrogen bond between residues TRP157 and SER183. While the interpretation of attention mechanisms remains an active area of research, this analysis provides an intuitive way to gain profound comprehension of the model’s functional mechanism. S^2^ALM showcases its ability to meaningfully highlight the key interaction sites within antibody structures, reaffirming the superiority of incorporating structural information during ALM pre-training.

### S^2^ALM accurately predicts antigen binding capacity

Establishing the precise binding specificity between antibodies and their target antigens is central to accelerating advancements in therapeutic antibody optimization and deepening our comprehension of the intricacies inherent to the immune response. Consequently, rapid and accurate evaluation of the antigen binding capacity stands as a pressing and fundamental requirement in the realm of antibody research and development. Antigen binding capacity prediction is a binary sequence classification task to determine binding capacity with the specific antigen, where each antibody is mapped to a neutralization or non-neutralization label. Specifically, we input antibody sequences into the pre-trained S^2^ALM to extract biological representations. The classification head (i.e., a linear projection layer) is added on top of the pre-trained model to output logits for identifying neutralization or non-neutralization classes. With a keen focus on the interaction between the target antigen human epidermal growth factor receptor 2 (HER2) and the clinically approved wild-type antibody trastuzumab, we compile a dataset of antibody-expressing sequences. These sequences are designed by replacing the original trastuzumab sequence with a myriad of variant CDR-H3 fragments from the heavy chain, as detailed in Refs. [18,26]. The resulting dataset boasts an impressive scale of 21,612 unique antibody sequences and applies the training/validation/test split of 15,128/3,242/3,242 (i.e., 70%/15%/15%). Our comparative analysis encompasses baselines of different types, including those pre-trained on protein sequences (i.e., ESM-1b [27] and MSA-1b [28]) and those pre-trained on antibody sequences (i.e., AbLang-H [17], AbLang-L [17], AntiBERTa [15], and EATLM [18]). Three classification metrics are utilized for evaluation. AUC is the area under the receiver operating characteristic curve, indicating the performance at all classification thresholds. F1 is the average weighted score of precision and recall. Matthews correlation coefficient (MCC) is the coefficient between true and predicted values.

We report the evaluation results of the proposed S^2^ALM and various baselines in Table 1 (left). On the one hand, ESM-1b and MSA-1b yield relatively inferior performance due to the domain knowledge gap between proteins and antibodies. On the other hand, among baselines pre-trained on antibody sequences, AbLang-H outperforms AbLang-L and AntiBERTa, indicating the advantage of separate training for heavy and light chain sequences. However, the true standout in these evaluations is S^2^ALM, which surpasses all baselines and sets a new state-of-the-art performance in this task. Such accomplishment underscores the capacity of S^2^ALM to transcend traditional sequence-based models in antigen binding prediction.

**Table 1.** Evaluation performance on antigen binding capacity prediction, B cell maturation analysis, and antibody paratope prediction tasks. The best experimental results are highlighted in bold. S^2^ALM achieves considerable improvements across all 3 antibody-specific tasks. This reveals the potential of S^2^ALM as the antibody-specific foundation model constructed for comprehensive antibody representation learning.

Method	Antigen binding prediction			Maturation analysis	Antibody paratope prediction		
	AUC	F1	MCC	ACC	AUC	F1	MCC
ESM-1b [27]	0.917	0.854	0.689	0.503	0.886	0.669	0.547
MSA-1b [28]	0.921	0.857	0.689	0.416	0.887	0.679	0.557
AbLang-H [17]	0.918	0.861	0.704	0.570	0.878	0.674	0.546
AbLang-L [17]	0.917	0.856	0.682	0.546	0.882	0.680	0.553
AntiBERTa [15]	0.918	0.843	0.678	0.565	0.879	0.690	0.559
EATLM [18]	0.922	0.862	0.699	0.581	0.887	0.698	0.575
S^2^ALM	**0.931**	**0.868**	**0.705**	**0.588**	**0.893**	**0.708**	**0.583**

### S^2^ALM precisely distinguishes B cell maturation states

B cells occupy a central position in the immune system’s protective arsenal, due to their distinctive ability to generate antibodies. In the human body, these antibodies serve as a vital line of defense against invading pathogens [7]. Therefore, exploring and analyzing the process of B cell antibody maturation remains crucial, enhancing our understanding of the intricate mechanisms that unfold during immune system evolution (i.e., a critical biological process affecting the function and antigen binding specificity of antibodies) [29,30]. B cell maturation analysis is a 6-category classification task. Its core objective lies in accurately distinguishing the maturation states of B cell antibody sequences. Each antibody sequence belongs to one of {*immature, transitional, mature, plasmacytes, memory IgD+, memory IgD-*} states. Accomplishing this necessitates a model capable of learning an evolution-aware representation sensitive to different B cell maturation states, with a linear projection layer serving as the classification head. We utilize a total of 88,094 antibody sequences from [18,31] with 6 maturation states and apply the default data split. The evaluation metric is accuracy (ACC), calculating the ratio of correct predictions.

Table 1 (center) demonstrates the findings of model ability to distinguish between multiple B cell mature states. For the baselines pre-trained on protein sequences, both ESM-1b [27] and MSA-1b [28] present inferior performance. Even though armed with evolution-aware representations learned from multiple protein sequence alignments, MSA-1b performs poorly in this antibody maturation related task. Such phenomenon suggests that there is a gap between protein and antibody domains. For those pre-trained on antibody-specific sequences (i.e., AbLang-H [17], AbLang-L [17], AntiBERTa [15], and EATLM [18]), they deliver promising results in this task. Notably, S^2^ALM, pre-trained on both antibody sequences and structures, achieves a substantial leap forward and surpasses all baselines by a large margin. The evaluation result highlights the efficacy of S^2^ALM in improving the comprehension of B cell maturation and immune evolutionary mechanisms.

### S^2^ALM thoroughly identifies antibody paratopes

In immunology research, identifying the amino acids in immunoglobulins that specifically bind to antigens (i.e., the antibody paratope) is one of the most crucial and challenging tasks [32]. The antibody paratope, usually composed of multiple amino acids in the complementarity-determining regions, forms the antibody binding site, which is essential for antigen interactions in the immune response [33]. Antibody paratope prediction could significantly help to enhance our understanding of the binding mechanisms of therapeutic antibodies. It is a binary token labeling task that identifies specific binding positions within antibody sequences, assigning a 0/1 label to each amino acid residue. We add a linear projection layer atop the pre-trained model to act as the prediction head. For paratope prediction, we utilize 2 antibody datasets sourced from Refs. [15,18], which comprise 277 and 900 antibody sequences annotated with token-wise paratope regions. Numerous baseline models are utilized, including Sapiens-H [34], ProtBERT [35], AntiBERTa [15], AntiBERTy [16], ESM-1b [27], ESM-2 [19], BALM [9], MSA-1b [28], AbLang-H [17], AbLang-L [17], and EATLM [18]. Experimental metrics used to evaluate the prediction capability include AUC, F1 score, and MCC.

Table 1 (right) reports results on antibody paratope prediction benchmark [18], and we observe that S^2^ALM achieves considerable improvements while other baselines show no significant difference in performance. Fig. 3D depicts results on antibody paratope prediction benchmark [15], where S^2^ALM stands out by outperforming all other baselines across all criteria. The holistic experimental results showcase the superiority of our model in efficiently capturing features associated with antibody binding sites. This further highlights S^2^ALM’s high specificity, accuracy, and in-depth understanding of antibody complex binding mechanisms.

### S^2^ALM accurately predicts antigen–antibody binding affinity

**Fig. 4.**
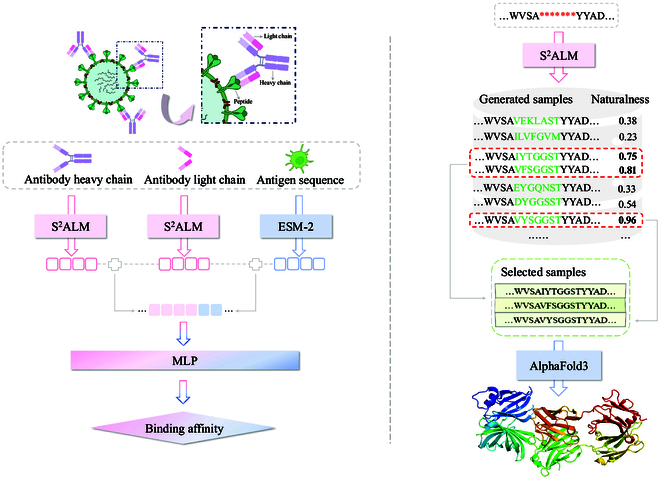
Overview of the S^2^ALM experimental workflow. Left: Detailed architecture of antigen–antibody binding affinity prediction. S^2^ALM is applied to extract antibody features, while ESM-2 is used for antigen feature encoding. The encoded features are concatenated and input into the MLP model to predict binding affinity. Right: Elaborate workflow of computational antibody design. S^2^ALM generates antibodies via sequence infilling, retaining top 3 samples with the highest naturalness. AlphaFold3 is incorporated for predicting 3D structures of antigen–antibody complexes.

Therapeutic antibody development has become an increasingly popular strategy for drug discovery, but still involves intensive laboratory experiments [36]. Recently, deep learning methods have emerged as a promising approach to accelerate such process by identifying high-affinity antibody candidates [36,37]. The property of binding affinity, which quantifies the binding strength between antigen–antibody pairs, plays a critical role in antibody development and optimization. To assess the model’s performance in predicting the binding affinity, we first utilize 2 datasets, 14H and 14L [38], which contain abundant antibodies labeled with binding affinity values. Within these 2 datasets, the affinity values quantify the binding strength of antibodies to a stable peptide in the HR2 region of SARS-CoV-2. To further expand the diversity of binding antigens and thereby enhance both the comprehensiveness and reliability of this experiment, we also include the BioMap [39] dataset for the evaluation of binding affinity prediction. The BioMap dataset contains 1,706 antigen–antibody pairing data with labeled binding affinity values, in which most antibodies stem from human and mouse sources. As for the experiment execution, the 14H, 14L, and BioMap datasets are each divided into the training/validation/test set according to the 80%/10%/10% split following He et al. [7]. Based on the test sets, we record experimental results for fair comparison with extensive baselines (i.e., Vanilla BERT [40], Ens-Grad [41], AbMAP [42], AntiBERTa2 [43], ESM-F [19], and A2binder [7]). In this binding affinity prediction task, we use the Pearson correlation and the Spearman correlation as evaluation metrics. Building upon pre-trained S^2^ALM, we extend and enhance the model architecture to enable the accurate learning of antigen–antibody binding rules. As illustrated in Fig. 4 (left), S^2^ALM is employed to extract features of antibodies, while ESM-2 [19] is introduced for the feature encoding of antigens. The antibody and antigen features extracted by pre-trained models are subsequently integrated through concatenation. Finally, the MLP (a multilayer perceptron) model utilizes the combined feature representation to predict binding affinity.

Table 2 illustrates the performance comparison of models in predicting the antigen–antibody binding affinity. S^2^ALM consistently achieves state-of-the-art performance in Pearson correlation and Spearman correlation metrics. We observe that S^2^ALM significantly surpasses all baselines, except for the BioMap dataset, where it achieves the same Spearman correlation as A2binder. The experimental results further verify the heightened precision of S^2^ALM in predicting the antigen–antibody binding affinity. By integrating both sequence and structure data for large-scale pre-training, S^2^ALM effectively captures the specific functional information of antibodies. We believe that S^2^ALM has the great potential to serve as the antibody-specific foundation model to promote the therapeutic antibody development in real-world scenarios.

**Table 2.** The performance comparison on antigen–antibody binding affinity prediction tasks. We highlight the best experimental results in bold. S^2^ALM achieves state-of-the-art performance across all evaluation metrics on the 3 antibody datasets (i.e., 14H, 14L and BioMap).

Method	14H		14L		BioMap	
	Pearson	Spearman	Pearson	Spearman	Pearson	Spearman
Vanilla BERT [40]	0.594 (0.021)	0.480 (0.025)	0.607 (0.024)	0.611 (0.027)	0.492 (0.036)	0.498 (0.037)
Ens-Grad [41]	0.601 (0.016)	0.476 (0.023)	0.637 (0.019)	0.645 (0.023)	0.645 (0.031)	0.664 (0.033)
AbMAP [42]	0.606 (0.015)	0.510 (0.015)	0.674 (0.012)	0.685 (0.016)	0.637 (0.027)	0.673 (0.029)
AntiBERTa2 [43]	0.623 (0.011)	0.545 (0.008)	0.673 (0.013)	0.684 (0.012)	0.633 (0.022)	0.670 (0.031)
ESM-F [19]	0.634 (0.007)	0.516 (0.010)	0.674 (0.011)	0.681 (0.014)	0.628 (0.028)	0.644 (0.024)
A2binder [7]	0.642 (0.012)	0.553 (0.011)	0.683 (0.010)	0.688 (0.015)	**0.701 (0.024)**	0.746 (0.025)
S^2^ALM	**0.650 (0.013)**	**0.558 (0.014)**	**0.687 (0.015)**	**0.693 (0.017)**	**0.701 (0.022)**	**0.749 (0.028)**

### S^2^ALM enables computational antibody CDR design

Given the extraordinary performance on antibody understanding tasks related to the evolutionary process and functional mechanism, we additionally incorporate a novel generation task to further evaluate the model’s capability of antibody generation and optimization. The complementarity-determining region (CDR), also known as the hypervariable region, primarily determines the antibody’s affinity and specificity for its target. Given the crucial role of CDRs, computational antibody design primarily focuses on generating these regions. Specifically, antibody CDR design is a sequence infilling task to generate masked CDRs (more exactly, CDR-H3) based on the contextualized representation. For antibody CDR design, the dataset curated by Jin et al. [44] follows the training/validation/test split of 2,282/291/291 samples derived from the Coronavirus Antibody Database (CoV-AbDab) [45]. CoV-AbDab is a public database documenting molecular information (sequences and structures) of all published and patented antibodies and nanobodies that can bind to coronaviruses, including SARS-CoV-2, SARS-CoV-1, and MERS-CoV [45]. During this experiment, we mask the CDRs in both 1D and 3Di antibody sequences for S^2^ALM to reconstruct. To benchmark the quality of antibody sequences generated by S^2^ALM, we employ ProtBERT [35], AbLang-H [17], and ReprogBERT [22] for comparison. Three metrics are utilized for holistic evaluation. Perplexity (PPL) measures how well the language model predicts the generative tokens. Here, we exploit off-the-shelf ProGen [46] for calculation following Melnyk et al. [22]. Lower values signify better performance, indicating stronger naturalness of the generated CDRs. Amino acid recovery (AAR) is computed for the specific sequence region of interest, measuring the percent of the exact matches between ground truth and sampled sequences. The higher the AAR, the more accurate the recovery. Diversity (DIV) computes the complement of the average recovery of all pairwise comparisons using sampled CDRs. A higher value corresponds to an increased dissimilarity among the samples themselves.

The evaluation results are shown in Fig. 3B. In spite of the high AAR, ProtBERT struggles in low generation diversity. AbLang-H and ReprogBERT are particularly talented in generation diversity but demonstrate relatively high perplexity. In contrast, S^2^ALM achieves substantially high AAR and the lowest perplexity while maintaining the comparable high diversity to ReprogBERT, accomplishing the balance of all 3 generative metrics. Leveraging the CDR infilling paradigm, S^2^ALM successfully generates CDR loop libraries with improved in silico developability profiles. This strongly indicates that S^2^ALM could serve as an effective and insightful tool for computational antibody design, benefiting the biomedical research community.

### 3D structural visualization

To further manifest the advantages of incorporating structural information during ALM pre-training, we conduct 3D visualization of antigen–antibody complexes generated by S^2^ALM. The thorough workflow for antibody sequence generation and structure visualization is depicted in Fig. 4 (right). Based on a holistic antigen–antibody complex dataset of 60 antigen–antibody pairs constructed by Adolf-Bryfogle et al. [47], S^2^ALM designs antibody CDR-H3 via sequence infilling. For each antigen–antibody complex, 100 CDR-H3 samples are generated and combined with standard framework regions to obtain 100 full-length antibody heavy chains. We select the top 3 antibody heavy chains with the highest naturalness using ProGen2 [48], following the standard protocol [7,22]. Subsequently, AlphaFold3 [25], highly capable of predicting the antigen–antibody joint structure, is applied to generate the 3D structures of complexes comprising antigen and generated antibodies (including antibody heavy chains and corresponding light chains). Evaluation metrics of the folded 3D structure are the predicted template-modeling (pTM) score, the interface pTM (ipTM), and the predicted local distance difference test (pLDDT). Moreover, to facilitate a thorough comparison, we involve a variety of language models: ESM-2 [19], pre-trained on general protein sequences; SaProt [49], which harnesses both protein sequences and structures for pre-training; and 2 dedicated ALMs, AntiBERTa [15] and AbLang-H [17]. The structural evaluation results through AlphaFold3 are illustrated in Fig. 3E. SaProt and AbLang-H stand out among all baselines, verifying the advantages of injecting structural information and promoting antibody-specific learning. S^2^ALM consistently achieves the optimal pTM, ipTM, and pLDDT scores compared to other methods, demonstrating that the generated antibodies are more likely to target the corresponding antigens and form stable binding complexes. Furthermore, we visualize the generated 3D complex structures targeting 3 clinically important pathogens (i.e., Vaccinia virus, *Neisseria meningitidis*, and Influenza B virus). Fig. 3F depicts some visualization examples. The stable and regular 3D structures of designed antigen–antibody complexes fully exhibit the superior generative capability of S^2^ALM, which pioneeringly incorporates 3D structural information into ALM pre-training.

## Discussion

Antibodies are vital proteins produced by the immune system to safeguard against and combat with a variety of diseases [1]. There are abundant clinical applications of antibody analysis in therapeutic drug discovery and finding novel diagnostics [6,7,15,50]. Most analyses have focused on comparing high-level differences between cohorts to facilitate antibody understanding, such as number of somatic hypermutations, isotype subclass usage, and V(D)J gene segment usage. With the advancements of machine learning techniques, it has been verified in various studies that transformer-based LLMs significantly promote the understanding and decoding of biological language [6,7,10,51,52]. The biological function of an antibody is directly determined by its 3D structure; thus, the modalities of sequence and structure should be tightly coupled to generate a comprehensive representation of the antibody. However, existing works fall short in effectively injecting structural information during antibody pre-training, which substantially hinders the further progress.

In this study, our endeavor herein offers an antibody-specific foundation model that posits the potential to facilitate comprehensive antibody understanding and generation. Extensive machine learning paradigms and techniques are utilized in developing S^2^ALM. To our best knowledge, S^2^ALM stands out as the first ALM to integrate hybrid information of 1D sequences and 3D structures during large-scale pre-training. S^2^ALM extracts comprehensive and meaningful antibody representations, uncovering inherent patterns and properties of functional specificity, biological species, and evolutionary isotypes. Additionally, as a supplement to interpretability analysis, S^2^ALM precisely captures crucial interaction sites within 3D structures. Evaluated on diverse computational scenarios, S^2^ALM exhibits a state-of-the-art performance in antibody representation learning: accurately predicting antigen binding capacity, precisely distinguishing B cell maturation states, thoroughly identifying antibody paratopes, and accurately predicting antigen–antibody binding affinity. To further explore the potential applications, we employ S^2^ALM for computational antibody design leveraging the CDR infilling paradigm. The generated stable and regular 3D structures further indicate the superior generative capability of S^2^ALM. We hold the expectation that our work will usher in a new era of antibody multi-level pre-training via explicitly modeling the profound interrelation between sequences and structures, fostering the development of therapeutic antibody drugs in real-world scenarios.

While our work demonstrates promising and outstanding results in extensive antibody-specific downstream tasks, there are still some areas for improvement and future exploration: (a) Due to computational constraints, the model size of S^2^ALM may not have reached its maximum capacity. (b) Although we circumnavigate such constraints by introducing extra protein structures in our hierarchical pre-training paradigm, the limitation of antibody structure data cannot be overlooked. In the future, we eagerly anticipate the emergence of large-scale antibody structure databases with sufficient experimentally determined 3D structures. Such efforts will fill the void of large-scale antibody structure corpus, thereby fueling multi-level antibody pre-training. (c) The performance of structural pre-training heavily depends on Foldseek [20], which aims to balance search efficiency and encoding accuracy. Thus, there is still room for improving the representation capability of all methods building upon Foldseek, including ours.

To conclude, this paper documents our efforts to build a comprehensive antibody-specific foundation model to represent the intricacies of the biological world. The capabilities demonstrated by S^2^ALM exhibit considerable promise and underscore several areas in antibody understanding and generation that necessitate substantial advancements. Such a multi-level pre-trained foundational model, integrating antibody sequential and structural information, will prove immensely valuable in accelerating immunotherapy development and enhancing our comprehension of biological phenomena.

## Materials and Methods

### Pre-training data

**Fig. 5.**
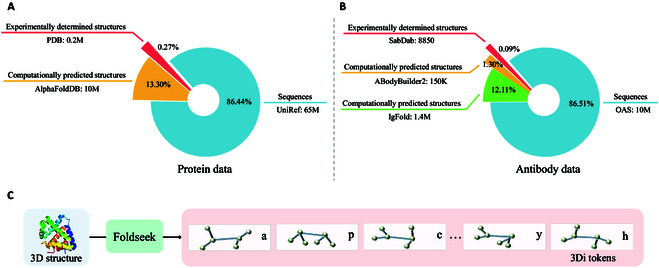
Illustrations of compositional ratios of the pre-training data and the structural encoding protocol. (A) The protein data contains 3 parts: sequences, experimentally determined structures, and computationally predicted structures. (B) The antibody data contains 4 parts: sequences, experimentally determined structures, and computationally predicted structures from ABodyBuilder2 [57] and IgFold [58]. (C) Efficient encoding protocol of 3D structures. Foldseek [20] is employed to discretize the target 3D structure into the 3Di sequences, deciphering 3Di states that describe the tertiary interaction between a residue and its nearest neighbor.

In the field of antibody pre-training, there is an abundance of sequential data, whereas the quantity of structure data remains limited, especially those determined by experiments. To compensate for the inadequacy of experimentally determined antibody structures, we additionally introduce computationally predicted antibody structures and general protein structures for comprehensive large-scale pre-training. Eventually, our holistic pre-training data encompass various levels and span multiple domains, including 75 million 1D sequences and 11.7 million 3D structures from protein and antibody domains. Fig. 5A and B presents compositional ratios of various parts in the pre-training dataset, and details of each part are as follows.

#### Protein sequence and structure data

Extensive protein sequence and structure data are incorporated for general sequence-structure learning. Specifically, for the primary structural information (i.e., sequential information), 65 million protein sequences are derived from UniRef50 [53], which is a clustering of UniRef90 seed sequences at 50% sequence identity. Moreover, to alleviate the insufficiency of antibody structure data, the extra protein structure data are incorporated. For the secondary and tertiary structural information, we utilize 0.2 million experimentally determined general protein 3D structures sourced from the PDB [54] and 10 million computationally predicted protein 3D structures from the AlphaFold Protein Structure Database (AFDB) [55].

#### Antibody sequence data

The pre-training antibody sequence data come from the OAS database [23], which contains over 2.4 billion unpaired sequences and 3 million paired sequences of antibody heavy and light chains. The OAS database boasts a rich collection of antibody sequences from 825 unique subjects spanning across 6 different species, where humans account for 88% and mice account for 11%. We downloaded the OAS database of unpaired and paired antibody sequences on 2024 January 11, and pre-processed the antibody sequence data by filtering, cleaning, and clustering. First, all duplicate antibody sequences were filtered out and removed. Additionally, we excluded antibody sequences containing unusual residues (selenocysteine and pyrrolysine), along with those having any framework region shorter than IMGT defined. Furthermore, to mitigate data leakage, we clustered antibody sequences at 70% sequence identity over the whole sequence using the MMseq2 algorithm [56]. Alignment coverage in MMseq2 was calculated with respect to the target sequence (“cov-mode 1”), with all other parameters set to their default values. Ultimately, the antibody sequence data comprise 43,551,946 unique antibody sequences, from which we randomly select 10 million sequences for training.

#### Antibody structure data

Given the central tenet that antibody structure substantially governs its function, we innovatively explore to inject antibody-specific structural knowledge into ALMs. To achieve this, we collect experimentally determined antibody structure data based on the Structural Antibody Database [59], which is composed of approximately 10 thousand antibody structures retrieved from the PDB [54]. Considering the limited quantity of experimentally determined antibody structures, we seek antibody structure prediction models for assistance to provide ample computationally predicted structures. Concretely, approximately 150 thousand antibody structures predicted by ABodyBuilder2 [57] and 1.4 million antibody structures predicted by IgFold [58] are incorporated for pre-training. Overall, the aforementioned 3 data sources collectively contribute to antibody structure learning.

### Structural encoding technique

Foldseek [20] is a computational tool for fast and accurate protein structure search, merging features such as amino acid spatial angles, distances, and sequence positions. It utilizes the vector quantized-variational autoencoder (VQ-VAE) model [60] to encode the 3D protein structure as distinct and information-rich 3Di tokens. Each amino acid is assigned a token based on its distance and relative position to the nearest amino acids in the folded protein structure. Foldseek achieves this transformation through the identification of the nearest neighbors and the extraction of distinctive features for individual residues [49]. In this paper, we introduce Foldseek to accomplish the efficient encoding of 3D structures, as depicted in Fig. 5C. Concretely, we adopt Foldseek with the default setting of 20 3Di tokens. Aligning all residue sites, protein and antibody 3D structures are transformed into 3Di sequences (i.e., 1D sequences storing 3D structural information). Such technique fills the void in effective encoding of structure data and enables S^2^ALM to handle sequence-structure multi-level information in a hybrid and unified manner, promoting comprehensive antibody representation learning.

### Hierarchical pre-training paradigm

#### Stage I: General sequence-structure learning

In pre-training stage I, we first tokenize the sequences and structures of proteins by an innovative multi-level vocabulary $F=V_{1}+V_{2}$. The original sequence alphabet $V_{1}$ consists of 20 standard amino acids. A protein or antibody sequence can be denoted as $s=\left\{s_{1},\;s_{2},\ldots ,\;s_{n}\right\}$, where $s_{i}\in V_{1}$ represents the 1D residue token at the *i*th site. Building upon the concept of Foldseek [20], the newly built structure alphabet $V_{2}$ utilizes 20 distinct 3Di tokens to accomplish the generation of pseudo structural sequences (i.e., 3Di sequences). A protein or antibody structure can be denoted as $a=\left\{a_{1},\;a_{2},\ldots ,\;a_{m}\right\}$, with $a_{j}\in V_{2}$ indicating the 3Di token at the *j*th site.

Building on the multi-level vocabulary, we obtain 1D and 3Di sequences and feed them into the model alternately. During pre-training stage I, we train S^2^ALM using the BERT-style masked language modeling (MLM) objective [40] to integratively learn from the 1D and 3Di sequences, enabling support for both sequence-level and structure-level tasks:

$$L_{1D-\mathrm{MLM}}=E\left[\sum_{i\in M}-\text{log}\;p\left(s_{i}|s_{/M}\right)\right],$$ (1)

$$L_{3\mathrm{Di}-\mathrm{MLM}}=E\left[\sum_{i\in M}-\text{log}\;p\left(a_{i}|a_{/M}\right)\right],$$ (2)

where M is randomly masked positions and $s_{/M},a_{/M}$ denote the masked sequence where all masked positions have been replaced by the [MASK] token.

Specifically, for each 1D or 3Di sequence, 15% of the corresponding tokens are randomly masked and then predicted based on the remaining contextualized representation. Consequently, the loss function $L_{I}$ in pre-training stage I is formulated as follows, where $L_{1D-\mathrm{MLM}}$ stands for 1D sequences and $L_{3\mathrm{Di}-\mathrm{MLM}}$ stands for 3Di sequences.

$$L_{I}=L_{1D-\mathrm{MLM}}+L_{3\mathrm{Di}-\mathrm{MLM}}.$$ (3)

Pre-training stage I endows the model with the capability to simultaneously identify both 1D and 3Di sequences. Furthermore, the efficient utilization of protein data in pre-training stage I effectively alleviates issues arising from insufficient antibody structure data. Due to the powerful generalization ability of large language models, the global structural constraints from proteins learned in stage I set the foundation for antibody-specific learning of sub-domain local constraints in stage II.

#### Stage II: Antibody-specific multi-level learning

After pre-training stage I, S^2^ALM has thoroughly comprehended 1D and 3Di sequences across the general protein domain. Subsequently in pre-training stage II, we can primarily focus on multi-level representation learning in the target antibody sub-domain. To better absorb comprehensive knowledge of antibody sequences and structures, exploring new pre-training mechanisms is worthwhile. Two multi-level learning objectives are introduced to inject different granularities of antibody-specific sequential and structural information into an ALM: sequence-structure matching (SSM) and cross-level reconstruction (CLR). The customized learning objectives facilitate the extraction of complex patterns and interdependency inherent in antibody sequences and structures.

SSM captures the coarse-grained alignment between antibody sequential and structural information. It is a binary classification task to predict whether a sequence-structure pair is matching or unmatching, as depicted in Fig. 1C. The model extracts representations of the corrupted antibody 1D and 3Di sequences and then exploits a linear layer to make classification on their matching relationships. SSM optimizes the following loss function:

$$L_{\mathrm{SSM}}=\sum_{\left(\tilde{s},\;\tilde{a}\right)\in \left(\tilde{S,\;A}\right)}H\left[y\left(\tilde{s},\;\tilde{a}\right),p(E_{\theta }(\tilde{s},\;\tilde{a}))\right],$$ (4)

where $\left(\tilde{S,\;A}\right)$ is the corrupted antibody dataset containing matched and unmatched sequence-structure pairs and *H* denotes the cross entropy loss function. $E_{\theta }$ and y represent parameterized encoder and matching ground truth, respectively.

CLR focuses on improving fine-grained understanding in antibody sequence-structure pre-training, which differs in reconstruction conditions from MLM in the “Stage I: General sequence-structure learning” section. Concretely, the paired antibody 1D and 3Di sequences are separately encoded by the model. Then, we randomly replace 15% of tokens with the [MASK] token in the 1D or 3Di sequence, while keeping the other level sequence unmasked. Eventually, as illustrated in Fig. 1D, hybrid information from both 1D and 3Di sequences serves as the CLR condition to make token-level predictions through a linear layer. CLR encourages the model to recover the corrupted 1D or 3Di sequences based on information from both levels, explicitly capturing the interrelated mechanism between antibody sequences and structures:

$$L_{1D-\mathrm{CLR}}=E\left[\sum_{i\in M}-\text{log}\;p\left(s_{i}|s_{/M};a\right)\right],$$ (5)

$$L_{3\mathrm{Di}-\mathrm{CLR}}=E\left[\sum_{i\in M}-\text{log}\;p\left(a_{i}|s;a_{/M}\right)\right],$$ (6)

where M is randomly masked positions in 1D or 3Di sequences. $L_{1D-\mathrm{CLR}}$ and $L_{3\mathrm{Di}-\mathrm{CLR}}$ subsequently measure the reconstruction costs of 1D and 3Di sequences. Therefore, the ultimate loss function $L_{\mathrm{II}}$ in pre-training stage II is formulated as:

$$L_{\mathrm{II}}=L_{\mathrm{SSM}}+L_{1D-\mathrm{CLR}}+L_{3\mathrm{Di}-\mathrm{CLR}}$$ (7)

With the groundwork laid in pre-training stage I, the tailored pre-training objectives in stage II facilitate S^2^ALM to effectively integrate antibody sequential and structural information, modeling comprehensive antibody representations. Overall, within this hierarchical pre-training paradigm, these 2 pre-training stages are complementary and indispensable to each other. Their synergy makes S^2^ALM a powerful antibody foundation model, further fostering holistic antibody understanding and generation. (See thorough pre-training details in Note S3.)

## Data Availability

The source code and data are freely available at https://github.com/KDurant-123/S2ALM.

## Supplementary Materials

Supplementary 1 Notes S1 to S6 Tables S1 and S2 References [61–70]
