# Supplementary material for: S2ALM: Sequence-Structure Pre-trained Large Language Model for Comprehensive Antibody Representation Learning
Source: Research (Wash D C). 2025 Aug 19;8:0721. doi: 10.34133/research.0721 (PMC12364524; doi:10.34133/research.0721)
Supplement: Supplementary 1 — Notes S1 to S6 Tables S1 and S2 References [61–70] [file research.0721.f1.pdf]

Supplementary Materials

Notes S1 to S6
Tables S1 and S2
References [61–70]

Note S1. Research Background

Utilizing massive amounts of text sequences, the transformer-based large-scale pre-training has become a widely adopted paradigm that demonstrates its remarkable capabilities in the field of Natural Language Processing (NLP) [36, 61]. Similar in the realm of life sciences, protein language models (PLMs) and antibody language models (ALMs), pre-trained on extensive biomolecular sequences, have showcased outstanding performance across a diverse array of tasks related to biological structures and functions [6, 15, 18, 19, 27]. Leveraging 1D sequential information, these PLMs and ALMs execute large-scale self-supervised pre-training to learn the language of life. However, since the three-dimensional spatial structures are directly relevant to biological functions, an intriguing and promising direction for their development is to incorporate extra 3D structure information into large-scale pre-training. Additionally, there have been some notable attempts in the protein domain but few in the antibody domain. It is primarily due to two critical challenges: (1) how to organize integrative pre-training data; (2) lack of efficient structural encoding technique. In this paper, we pay high attention to these two challenges and overcome them individually to pave the way for the training of S^2ALM. A holistic pre-training dataset is constructed, encompassing various levels and spanning multiple domains. And Foldseek in introduced to accomplish the efficient 3D structure encoding.

Note S2. Motivations of S^2ALM

Existing ALMs learn antibody representations primarily based on their 1D residue sequences. However, the 3D structures encompass intricate spatial geometric knowledge and are directly relevant to antibody functions, which demonstrates the full potential to enhance antibody representation learning. In this paper, we organize pre-training data including antibody sequences and structures and propose to integrate sequential and structural information during pre-training. Since the scale of available antibody structure data is relatively small compared to the protein data, the additional protein data comprising sequences and structures is introduced as a compensation, assisting the ALM to comprehensively incorporate structural information. By taking advantage of information from multiple levels and additional domains, we execute hierarchical pre-training to obtain an ALM effective on various antibody specific downstream tasks.

Note S3. Pre-training Details

Building on the architecture of ESM-2 [19], S^2ALM has 650 million trainable parameters. We incorporate 33 transformer encoder blocks, each containing 20 self-attention heads. And the hidden dimension is set to 1280. We exploit layer normalization to stabilize and speed up the training process

by reducing internal covariate shift. The Rotary Position Embedding (RoPE) is employed to supply token positional information. Additionally, we truncate all training sequences to a maximum length of 1024. Sequences of length less than 1024 are padded, and padded tokens are excluded from the loss computation. By utilizing token-type encoding, the model with mixed training distinguishes 1D and 3Di sequences, assigning 0 to 1D sequences and 1 to 3Di sequences.

Following BERT [36] and ESM-2 [19], during training of Masked Language Modeling (MLM), 15% of tokens in each batch are randomly selected and masked. For these selected tokens, 80% are substituted with the [MASK] special token, while 10% of them are replaced by random amino acids and the remaining 10% are left unchanged. For the training of Sequence-Structure Matching (SSM), we use an in-batch sampling strategy similar to CLIP [62] to corrupt the pairs of 1D and 3Di sequences. Given a mini-batch with N paired data, we construct an ensemble of N^2 1D and 3Di sequence pairs (including N correct pairings and $N^2 - N$ incorrect pairings) for model to predict whether they match or not. Regarding Cross-Level Reconstruction (CLR), we aim to reconstruct single-level information (*i.e.*, solely 1D sequence or 3Di sequence) based on both levels of information. Note that the MLM task on antibody 1D sequences is additionally interspersed in pre-training stage II. Such operation avoids catastrophic forgetting and ensures the preservation of sequential information when incorporating cross-level insights from antibody structures.

Our model is implemented using the PyTorch framework [63] and pre-trained on 32 NVIDIA Tesla V100 GPUs. For large-scale distributed training, we exploit the DeepSpeed ZeRO Stage 2 strategy [64]. The learning rate is 4×10^{-4} and 1×10^{-3} for pre-training stage I and II respectively. We also leverage mixed precision training for efficient pre-training. For training optimization, an AdamW optimizer is used with weight decay of 0.01. And we set a linear learning rate scheduler with 2,000 warmup steps. Eventually, we will make our codes, model weights, and the associated datasets openly available upon acceptance. These materials are expected to be valuable for both the computational and biological communities.

Note S4. Advantages over other LLMs

Table S1 presents distinct pre-training data types, objectives, downstream tasks. Earlier PLMs [19, 27, 35, 46] exclusively focus on protein sequences, which is similar to current ALMs [6, 7, 15–18] pre-trained solely on antibody sequences. Recently, an increasing number of PLMs [49, 65–69] have explored the incorporation of protein structures for pre-training, further enriching the pre-training data and obtaining powerful and generalized protein representations. Motivated by their promising endeavors and the significance of structural information, we propose to integrate antibody sequences and structures for multi-level pre-training. S^2ALM pioneeringly makes full use of sequences and structures across multiple domains within a hierarchical pre-training paradigm, significantly contributing to comprehensive antibody representation learning. Additionally, we customize two objectives to dig out the profound interconnections among biological multi-level information in antibody pre-training. Furthermore, S^2ALM is comprehensively evaluated in extensive experiments across multiple aspects of antibody applications.

Table S1: Different LLMs, pre-trained for protein and antibody representation learning, vary in their utilization of distinct pre-training data types, objectives and downstream tasks. To the best of our knowledge, S^2ALM is the first ALM to simultaneously incorporate protein sequences, antibody sequences, protein structures, antibody structures for large-scale pre-training, accomplishing comprehensive antibody representation learning. Additionally, two novel pre-training objectives are proposed to integrate sequential and structural information. Furthermore, the superiority of S^2ALM is holistically assessed on a variety of antibody-related experiments. CLM: causal language modeling; MLM: masked language modeling. CRD: coordinate prediction; ILM: infilling language modeling; AGP: ancestor germline prediction; MPP: mutation position prediction. SSM: sequence-structure matching; CLR: cross-level reconstruction.

**Model**	**LLM category**	**Pre-training data**				**Objective**	**Antibody specific downstream task**
		Sequence		Structure			
		Protein	Antibody	Protein	Antibody		
ProtBERT [35]	PLM	✓	✗	✗	✗	MLM	-
ESM-1b [27]	PLM	✓	✗	✗	✗	MLM	-
ESM-2 [19]	PLM	✓	✗	✗	✗	MLM	-
ProGen [46]	PLM	✓	✗	✗	✗	CLM	-
PromptProtein [65]	PLM	✓	✗	✓	✗	MLM+CRD	-
SaProt [49]	PLM	✓	✗	✓	✗	MLM	-
AntiBERTa [15]	ALM	✗	✓	✗	✗	MLM	functionality
AntiBERTy [16]	ALM	✗	✓	✗	✗	MLM	functionality & evolution
AbLang [17]	ALM	✗	✓	✗	✗	MLM	generation
IgLM [6]	ALM	✗	✓	✗	✗	ILM	functionality & generation
PALM[7]	ALM	✗	✓	✗	✗	MLM+CLM	generation
EATLM [18]	ALM	✗	✓	✗	✗	MLM+AGP+MPP	functionality & evolution
IgBERT, IgT5 [41]	ALM	✓	✓	✗	✗	MLM	functionality
S^2ALM stage I	ALM	✓	✗	✓	✗	MLM	functionality & evolution & generation
S^2ALM stage II	ALM	✗	✓	✗	✓	MLM+SSM+CLR	
S^2ALM	ALM	✓	✓	✓	✓	MLM+SSM+CLR	

Note S5. Ablation Study

To evaluate effectiveness of the proposed pre-training objectives, which are tailored for integrating the information from both antibody 1D sequences and 3D structures, we conduct a holistic ablation analysis on multi-type antibody related tasks. The ablation results are reported in Table S2. Overall, the performance will decay if any one of the constructed objectives is absent, indicating that both SSM and CLR are essential and advantageous to learn comprehensive antibody representations. In particular, for antibody paratope prediction task, the absence of SSM leads to a more significant performance drop compared to the lack of CLR. This is likely because the matching prediction of 1D and 3Di sequences efficiently injects information from antibody 3D structures that directly determines the spatial binding specificity with antigens. Furthermore, we notice that removing both SSM and CLR for pre-training yields the worst performance among all the ablation experiments. Such phenomenon confirms the superiority of incorporating structural information during the pre-training process and provides more insights into our method.

Note S6. Related Work

Encouraged by the success of PLMs in protein representation learning, series of works seeks to conduct large-scale pre-training for antibody specific representation learning. AntiBERTy [16] pioneeringly executes the antibody specific pre-training using 558 million antibody sequences. AntiBERTa [15] proposes an ALM proficient at the paratope prediction task. AbLang [17] separately

Table S2: Ablation results of S^2ALM on multi-type antibody related tasks. We primarily validate the effectiveness of designed objectives during S^2ALM pre-training. **Bold** indicates the best results. The performance will decay if any one of the constructed pre-training objectives is absent, demonstrating that all the objectives provide significant benefits.

	**Antigen Binding Prediction**			**Maturation Analysis**	**Antibody Paratope Prediction**		
	AUC	F1	MCC	ACC	AUC	F1	MCC
Full losses	**0.931**	**0.868**	**0.705**	**0.588**	**0.893**	**0.708**	**0.583**
w/o SSM	0.916	0.858	0.681	0.576	0.884	0.677	0.566
w/o CLR	0.917	0.860	0.684	0.573	0.889	0.695	0.570
w/o SSM&CLR	0.902	0.847	0.664	0.562	0.879	0.671	0.552

trains AbLang-H and AbLang-L using the heavy-chains and light-chains of antibody sequences to restore missing residues of antibody sequence data. IgLM [6], pre-trained on 558 million antibody sequences while conditioning on the chain type and species-of-origin, innovatively creates synthetic libraries by re-designing variable-length spans of antibody sequences. AbBERT [70] is trained by predicting masked amino acids in Complementarity Determining Regions (CDRs), which is customized for the antigen-specific antibody design task. EATLM [18] is the first ALM trying to inject antibody evolution information to language models and demonstrates good performance on the ATUE benchmark. To better leverage knowledge from the field of proteins, IgBERT and IgT5 [41] are two ALMs performing antibody sequence pre-training based on the initial weights of ProtBERT [35] and ProtT5 [35], and they facilitate the antigen-related binding energy prediction. Recently, PALM [7] demonstrates the remarkable capabilities of ALMs in both the accurate prediction of binding affinity and the diverse generation of antibody candidates. Compared to works in the general protein field [49, 65, 67–69], there has been limited exploration of integrating information of 1D sequences and 3D structures during large-scale pre-training for comprehensive antibody representation learning. AntiBERTa2-CSSP [39] exploits the contrastive learning technique to align the feature spaces of antibody sequences and structures, but the absence of a hybrid encoder and large-scale pre-training hinders further progress. In this paper, S^2ALM proposes a step towards integrating 1D sequences and 3D structures for large-scale pre-training, ultimately learning comprehensive antibody representations.

References

1. Taylor PC, Adams AC, Hufford MM, De La Torre I, Winthrop K, and Gottlieb RL. Neutralizing monoclonal antibodies for treatment of COVID-19. Nature Reviews Immunology 2021;21:382–93.

2. Ma X, Liang J, Zhu G, et al. SARS-CoV-2 RBD and Its Variants Can Induce Platelet Activation and Clearance: Implications for Antibody Therapy and Vaccinations against COVID-19. Research 2023;6:0124.

3. Li P, Yang Y, Wang Y, et al. Anti-TNFR2 Antibody-Conjugated PLGA Nanoparticles for Targeted Delivery of Adriamycin in Mouse Colon Cancer. Research 2024;7:0444.

4. Yin M, Zhou H, Zhu Y, et al. Synergy of GFlowNet and Protein Language Model Makes a Diverse Antibody Designer. In: *Proceedings of the AAAI Conference on Artificial Intelligence*. Vol. 39. 21. 2025:22164–72.
5. Mullard A. FDA approves 100th monoclonal antibody product. Nature reviews. Drug discovery 2021;20:491–5.
6. Shuai RW, Ruffolo JA, and Gray JJ. IgLM: Infilling language modeling for antibody sequence design. Cell Systems 2023;14:979–89.
7. He H, He B, Guan L, et al. De novo generation of SARS-CoV-2 antibody CDRH3 with a pre-trained generative large language model. Nature Communications 2024;15:6867.
8. Luo Y, Liu XY, Yang K, et al. Toward Unified AI Drug Discovery with Multimodal Knowledge. Health Data Science 2024;4:0113.
9. Jing H, Gao Z, Xu S, et al. Accurate prediction of antibody function and structure using bio-inspired antibody language model. Briefings in Bioinformatics 2024;25:bbae245.
10. Nguyen E, Poli M, Durrant MG, et al. Sequence modeling and design from molecular to genome scale with Evo. Science 2024;386:eado9336.
11. Mou M, Pan Z, Zhou Z, et al. A Transformer-Based Ensemble Framework for the Prediction of Protein-Protein Interaction Sites. Research 2023;6:0240.
12. Zhang Y, Guan J, Li C, et al. DeepSecE: A Deep-Learning-Based Framework for Multiclass Prediction of Secreted Proteins in Gram-Negative Bacteria. Research 2023;6:0258.
13. Zhou H, Yin M, Wu W, et al. ProtCLIP: Function-informed protein multi-modal learning. In: *Proceedings of the AAAI Conference on Artificial Intelligence*. Vol. 39. 21. 2025:22937–45.
14. Liu Z, Qian W, Cai W, et al. Inferring the Effects of Protein Variants on Protein-Protein Interactions with Interpretable Transformer Representations. Research 2023;6:0219.
15. Leem J, Mitchell LS, Farmery JH, Barton J, and Galson JD. Deciphering the language of antibodies using self-supervised learning. Patterns 2022;3:100513.
16. Ruffolo JA, Gray JJ, and Sulam J. Deciphering antibody affinity maturation with language models and weakly supervised learning. arXiv preprint arXiv:2112.07782 2021.
17. Olsen TH, Moal IH, and Deane CM. AbLang: an antibody language model for completing antibody sequences. Bioinformatics Advances 2022;2:vbac046.
18. Wang D, Ye F, and Zhou H. On Pre-trained Language Models for Antibody. In: *International Conference on Learning Representations*. 2023.
19. Lin Z, Akin H, Rao R, et al. Evolutionary-scale prediction of atomic-level protein structure with a language model. Science 2023;379:1123–30.
20. Van Kempen M, Kim SS, Tumescheit C, et al. Fast and accurate protein structure search with Foldseek. Nature Biotechnology 2024;42:243–6.
21. Van der Maaten L and Hinton G. Visualizing data using t-SNE. Journal of machine learning research 2008;9.

22. Melnyk I, Chenthamarakshan V, Chen PY, et al. Reprogramming pretrained language models for antibody sequence infilling. In: *International Conference on Machine Learning.* 2023.
23. Olsen TH, Boyles F, and Deane CM. Observed Antibody Space: A diverse database of cleaned, annotated, and translated unpaired and paired antibody sequences. Protein Science 2022;31:141–6.
24. Fagarasan S, Kinoshita K, Muramatsu M, Ikuta K, and Honjo T. In situ class switching and differentiation to IgA-producing cells in the gut lamina propria. Nature 2001;413:639–43.
25. Abramson J, Adler J, Dunger J, et al. Accurate structure prediction of biomolecular interactions with AlphaFold 3. Nature 2024:1–3.
26. Mason DM, Friedensohn S, Weber CR, et al. Optimization of therapeutic antibodies by predicting antigen specificity from antibody sequence via deep learning. Nature Biomedical Engineering 2021;5:600–12.
27. Rives A, Meier J, Sercu T, et al. Biological structure and function emerge from scaling unsupervised learning to 250 million protein sequences. Proceedings of the National Academy of Sciences 2021;118:e2016239118.
28. Rao RM, Liu J, Verkuil R, et al. MSA Transformer. In: *International Conference on Machine Learning.* 2021.
29. Ghraichy M, Niederhäusern V von, Kovaltsuk A, Galson JD, Deane CM, and Trück J. Different B cell subpopulations show distinct patterns in their IgH repertoire metrics. Elife 2021;10:e73111.
30. Meffre E, Casellas R, and Nussenzweig MC. Antibody regulation of B cell development. Nature Immunology 2000;1:379–85.
31. Mroczek ES, Ippolito GC, Rogosch T, et al. Differences in the composition of the human antibody repertoire by b cell subsets in the blood. Frontiers in immunology 2014;5:96.
32. Bashford-Rogers R, Bergamaschi L, McKinney E, et al. Analysis of the B cell receptor repertoire in six immune-mediated diseases. Nature 2019;574:122–6.
33. Kunik V, Ashkenazi S, and Ofran Y. Paratome: an online tool for systematic identification of antigen-binding regions in antibodies based on sequence or structure. Nucleic Acids Research 2012;40:W521–W524.
34. Prihoda D, Maamary J, Waight A, et al. BioPhi: A platform for antibody design, humanization, and humanness evaluation based on natural antibody repertoires and deep learning. MAbs 2022;14:2020203.
35. Elnaggar A, Heinzinger M, Dallago C, et al. ProtTrans: Toward Understanding the Language of Life Through Self-Supervised Learning. IEEE Transactions on Pattern Analysis and Machine Intelligence 2022;44:7112–27.
36. Devlin J, Chang MW, Lee K, and Toutanova K. BERT: Pre-training of Deep Bidirectional Transformers for Language Understanding. arXiv preprint arXiv:1810.04805 2019.
37. Liu G, Zeng H, Mueller J, et al. Antibody complementarity determining region design using high-capacity machine learning. Bioinformatics 2020;36:2126–33.

38. Singh R, Im C, Qiu Y, et al. Learning the language of antibody hypervariability. bioRxiv 2023:2023–4.

39. Barton J, Galson JD, and Leem J. Enhancing antibody language models with structural information. bioRxiv 2024:2023–12.

40. Li L, Gupta E, Spaeth J, Shing L, Bepler T, and Caceres RS. Antibody Representation Learning for Drug Discovery. arXiv preprint arXiv:2210.02881 2022.

41. Kenlay H, Dreyer FA, Kovaltsuk A, Miketa D, Pires D, and Deane CM. Large scale paired antibody language models. arXiv preprint arXiv:2403.17889 2024.

42. Engelhart E, Emerson R, Shing L, et al. A dataset comprised of binding interactions for 104,972 antibodies against a SARS-CoV-2 peptide. Scientific Data 2022;9:653.

43. Chen B, Cheng X, Geng Ya, et al. xTrimoPGLM: Unified 100B-Scale Pre-trained Transformer for Deciphering the Language of Protein. bioRxiv 2024.

44. Jin W, Wohlwend J, Barzilay R, and Jaakkola TS. Iterative Refinement Graph Neural Network for Antibody Sequence-Structure Co-design. In: *International Conference on Learning Representations*. 2022.

45. Raybould MIJ, Kovaltsuk A, Marks C, and Deane CM. CoV-AbDab: the Coronavirus Antibody Database. Bioinformatics 2021;37:734–5.

46. Madani A, Krause B, Greene ER, et al. Large language models generate functional protein sequences across diverse families. Nature Biotechnology 2023;41:1099–106.

47. Adolf-Bryfogle J, Kalyuzhniy O, Kubitz M, et al. RosettaAntibodyDesign (RAbD): A general framework for computational antibody design. Public Library of Science Computational Biology 2018;14:e1006112.

48. Nijkamp E, Ruffolo JA, Weinstein EN, Naik N, and Madani A. ProGen2: Exploring the boundaries of protein language models. Cell Systems 2023;14:968–978.e3.

49. Su J, Han C, Zhou Y, Shan J, Zhou X, and Yuan F. SaProt: Protein Language Modeling with Structure-aware Vocabulary. In: *International Conference on Learning Representations*. 2024.

50. Yin M, Zhou H, Zhu Y, et al. Multi-Modal CLIP-Informed Protein Editing. arXiv preprint arXiv:2407.19296 2024.

51. Zhu Y, Wu J, Li Q, et al. Bridge-IF: Learning Inverse Protein Folding with Markov Bridges. arXiv preprint arXiv:2411.02120 2024.

52. Shi Z, Deng R, Yuan Q, et al. Enzyme Commission Number Prediction and Benchmarking with Hierarchical Dual-core Multitask Learning Framework. Research 2023;6:0153.

53. Suzek BE, Wang Y, Huang H, McGarvey PB, Wu CH, and Consortium U. UniRef clusters: a comprehensive and scalable alternative for improving sequence similarity searches. Bioinformatics 2015;31:926–32.

54. Berman HM, Westbrook J, Feng Z, et al. The protein data bank. Nucleic Acids Research 2000;28:235–42.

55. Varadi M, Anyango S, Deshpande M, et al. AlphaFold Protein Structure Database: massively expanding the structural coverage of protein-sequence space with high-accuracy models. Nucleic Acids Research 2022;50:439–44.
56. Abanades B, Wong WK, Boyles F, Georges G, Bujotzek A, and Deane CM. ImmuneBuilder: Deep-Learning models for predicting the structures of immune proteins. Communications Biology 2023;6:575.
57. Ruffolo JA, Chu LS, Mahajan SP, and Gray JJ. Fast, accurate antibody structure prediction from deep learning on massive set of natural antibodies. Nature Communications 2023;14:2389.
58. Steinegger M and Söding J. Clustering huge protein sequence sets in linear time. Nature Communications 2018;9:2542.
59. Dunbar J, Krawczyk K, Leem J, et al. SAbDab: the structural antibody database. Nucleic Acids Research 2014;42:D1140–D1146.
60. Aaron vdO, Oriol V, and Koray K. Neural discrete representation learning. In: *Advances in Neural Information Processing Systems*. 2017.
61. Achiam J, Adler S, Agarwal S, et al. GPT-4 technical report. arXiv preprint arXiv:2303.08774 2023.
62. Radford A, Kim JW, Hallacy C, et al. Learning transferable visual models from natural language supervision. In: *International Conference on Machine Learning*. 2021:8748–63.
63. Paszke A, Gross S, Massa F, et al. PyTorch: An Imperative Style, High-Performance Deep Learning Library. In: *Advances in Neural Information Processing Systems*. 2019.
64. Rajbhandari S, Rasley J, Ruwase O, and He Y. ZeRO: Memory optimizations toward training trillion parameter models. In: *SC20: International Conference for High Performance Computing, Networking, Storage and Analysis*. 2020.
65. Wang Z, Zhang Q, Yu H, et al. Multi-level protein structure pre-training with prompt learning. In: *International Conference on Learning Representations*. 2023.
66. Chen C, Zhou J, Wang F, Liu X, and Dou D. Structure-aware protein self-supervised learning. Bioinformatics 2023;39:btad189.
67. Zhang Z, Xu M, Lozano A, Chenthamarakshan V, Das P, and Tang J. Enhancing protein language model with structure-based encoder and pre-training. In: *ICLR 2023-Machine Learning for Drug Discovery workshop*. 2023.
68. Lee Y, Yu H, Lee J, and Kim J. Pre-training Sequence, Structure, and Surface Features for Comprehensive Protein Representation Learning. In: *International Conference on Learning Representations*. 2023.
69. Heinzinger M, Weissenow K, Sanchez JG, Henkel A, Steinegger M, and Rost B. ProstT5: Bilingual Language Model for Protein Sequence and Structure. bioRxiv 2023.
70. Gao K, Wu L, Zhu J, et al. Pre-training Antibody Language Models for Antigen-Specific Computational Antibody Design. In: *ACM SIGKDD International Conference on Knowledge Discovery and Data Mining*. 2023.
